# Impact of COVID-19 Pandemic on the Self-Reported Frequency of Hospital Visits and Pediatric Care Outcomes in the Kingdom of Saudi Arabia

**DOI:** 10.7759/cureus.20958

**Published:** 2022-01-05

**Authors:** Naif Z Almutairi, Abdulaziz M Almutairi, Ibrahim S Alduhayshi, Jarallah F Alfarraj, Mohammed A Alrawsaa, Ahmad M Almazroa, Abdulelah M Almahfuth, Elsadig Y Mohamed, Abdullah M AlOlayan

**Affiliations:** 1 College of Medicine, Majmaah University, Al Majmaah, SAU; 2 College of Medicine, Department of Community Medicine and Public Health, Majmaah University, Al Majmaah, SAU; 3 College of Medicine, Department of Pediatrics, Majmaah University, Majmaah, SAU

**Keywords:** children, emergency department visits, pediatric care, ksa, covid-19

## Abstract

Introduction

Coronavirus disease 2019 (COVID-19) pandemic has influenced various aspects of health care and its outcomes. Several studies conducted on different age groups from different countries have reported a decrease in the frequency of hospital visits during the pandemic.

Aim

This study aimed to assess the self-reported effect of the COVID-19 pandemic on hospital visits and healthcare outcomes in the pediatric age group. We further aimed to assess the participants’ beliefs on the reasons for decreased frequency of hospital visits during the pandemic.

Methods

This was a quantitative analytical cross-sectional study. Data from the parents of children less than 14 years living in the Kingdom of Saudi Arabia (KSA) was collected using a self-administered structured questionnaire. The questionnaire included sociodemographic characteristics of the participants, self-reported frequency of hospital visits, and potential consequences on pediatric care outcomes during the pandemic. A total of 1,548 initial respondents filled the questionnaire, out of which only 1,311 had children aged less than 14 years. SPSS version 25 (IBM, New York, USA) was used for statistical analysis.

Results

We found that of the 1,311 eligible respondents, majority (75.1%) were mothers of the children while only 24.9% were fathers, and 34.4% of the respondents had two children less than 14 years. Majority (76.7%) of the respondents were from the central region and felt that the pandemic has reduced their frequency of hospital visits. Furthermore, majority (78.6%) of the respondents believed that the decreased hospital (emergency or clinic) visits did not have any negative effect on the health and care of their children. Moreover, 56.4% of the participants responded that lack of the need to visit the hospital during the pandemic was the reason for their decreased hospital visits frequency, and 51.6% were afraid of being infected by the virus. There was a significant association between decreased hospital visits and missing an appointment for vaccination, delayed diagnosis, deterioration of participants’ children’s condition, and running out of treatment and inability to refill.

Conclusion

This study was conducted to assess the impact of the COVID-19 pandemic on hospital visits and pediatric care outcomes in the KSA. We hypothesized that the pandemic has led to a reduction in pediatric hospital visits which might influence pediatric care outcomes.

We found that there was a decrease in the frequency of hospital visits. This decrease was attributed to the lack of the need to go to the hospital or to the fear of being infected by the virus. A significant association was found between the participants’ beliefs of the pandemic effect on hospital visits and its effect on the pediatric care outcomes.

## Introduction

Coronavirus disease 2019 (COVID-19) is an infectious disease that was first discovered in Wuhan, China at the end of December 2019. Severe acute respiratory syndrome coronavirus 2 (SARS-CoV-2) is a member of the coronaviruses (CoV) family that includes the Middle East respiratory syndrome (MERS-CoV) and SARS-CoV [1]. Most infected individuals have mild to moderate symptoms and recover without the need for hospitalization. The incubation period of the virus is 5 to 6 days, but it could take up to 14 days [2]. Globally, as of November 2, 2021, more than 247 million people were confirmed to have COVID-19 with over 5 million deaths [3], and in the Kingdom of Saudi Arabia (KSA), 548,666 people were confirmed to have COVID-19 with 8,796 deaths [4]. As a result of the increase in the number of cases and under the influence of the lockdown, there were multiple challenges from different aspects that the world had to encounter. These aspects are psychological, social, and economical. The psychological and social burden was evident by the increased number of anxiety, depression, and social isolation [5].

Due to the huge burden on the healthcare system that resulted from the pandemic, there was a decrease in the frequency of seeking medical care measured by hospital visits as reported by several studies. For example, a retrospective study conducted in the United States aimed to compare the number of ED visits per week in the last 4 weeks during the pandemic with that of the last 4 weeks before the pandemic. There was a significant decrease in the number of ED visits during the pandemic period with respect to the region variant [6]. Another observational study in the United States showed a gradual and significant decline in ED visits with an average of 250 and 167 visits per day before and after the announcement of the pandemic, respectively [7]. Regionally, a study conducted at Hamad medical corporation in Qatar demonstrated that the number of ED visits during the pandemic declined by 20-43% [8].

This decrease in the frequency of hospital visits involved all ages and all hospital services including pediatric age group visiting ED and outpatient clinics. A study conducted in Italy compared all visits during the strict lockdown period with visits during the same period in the prior 2 years. They found a significant decrease in the number of pediatric ED visits during the lockdown compared with the previous 2 years [9]. Another study in India also found a decline in pediatric ED and outpatient visits before and during the COVID-19 pandemic [10]. Moreover, a study that included data from 27 hospitals in the United States showed that the rates of pediatric ED visits decreased by 45.7% during the pandemic [11].

The decreased number of hospital visits during the pandemic can negatively impact healthcare outcomes. This impact can be of greater significance especially in the pediatric age group who require regular hospital visits, check-ups, and specialized care. Several studies addressed the impact of decreased hospital visits on pediatric care outcomes and had conflicting findings. For instance, a study conducted in Italy revealed that the decreased number of hospital visits did not influence the pediatric care outcomes as measured by the increases in the number of life-threatening cases or delay in the diagnosis [12]. In contrast, a study conducted in the UK reported that a delayed presentation with diabetes mellitus, being the most common, was observed by 32% of the pediatric consultants involved in the study [13]. Another large study in the United States involved 144 EDs and four urgent care clinics in 18 states and revealed that there was a decrease in the diagnosis of non-COVID-19 communicable diseases. In addition, they found that there was a decline in common but serious pediatric diseases, such as appendicitis, throughout the pandemic period [14].

Due to the importance of regular follow-up and appointment attendance for the pediatric age group to the pediatric clinic, we hypothesized that the COVID-19 pandemic might be associated with a decrease in hospital visits and, therefore, poor pediatric care outcomes.

## Materials and methods

Study design

This was a cross-sectional study on data collected using a self-administered structured questionnaire. The questionnaire included sociodemographic characteristics of the participants, self-reported frequency of hospital visits, and potential consequences on pediatric care outcomes during the pandemic.

Study population

The study population were children living in the KSA. The study area covered all regions of Saudi Arabia, namely, the central, western, eastern, northern, and southern regions.

Inclusion and exclusion criteria

In line with the research carried out by AlSowailmi et al. (2018) [15], only parents with children aged less than 14 years were included. Those with children aged more than 14 years and those unwilling to participate in the study were excluded.

Sample size

A trusted website www.raosoft.com was used to calculate the sample size [16]. The calculated minimum number was 385 with 95% CI. We received 1,548 filled questionnaires forms. However, out of 1,548 initial respondents, only 1,311 had children aged less than 14 years. Thus, the final sample size (N) was 1,311.

Data collection

A team of data collectors was hired for this study. A 3-day training was provided to all data collectors with the standard operating procedures for data collection from the prospective study respondents. The data collectors used social media platforms, namely, Facebook, Twitter, and WhatsApp groups for data collection from the respondents. For each social media platform, there were two data collectors assigned to choose the participants, based on the study inclusion criteria, in a systematic manner that they were trained on. Large groups from different regions of the KSA were specifically targeted to cover all areas and invite more participants. In total, 1,548 participants were willing to participate in the study. The structured questionnaire was developed using Google forms, along with the informed consent form. These 1,548 participants received a hyperlink, upon accessing the link, the participants were directed to a page on the Google forms containing all essential details that were provided in the informed consent form such as the aim of the study, respondents’ rights, confidentiality eligibility, types of information that they would provide, and significance of the study findings.

Ethical issues

Ethical approval was obtained from the Majmaah University Ethics Committee. All the participants provided their consent online before filling in the questionnaire. The informed consent form contained all essential information for the study participants to help them understand the purpose of the study and utilization of the results in making well-informed evidence-based health policies and strategies.

Statistical analysis

IBM Statistical Package for the Social Sciences (IBM, New York, USA) version 25 was used for statistical analyses. Descriptive statistics were presented using frequency distribution, percentages, means, and standard deviation. Charts were used for graphical descriptive presentation. Cross-tabulations and the chi-square test of association were used to examine the respondent’s characteristics and their perception of the pandemic effect on hospital visits and were used for other inferential analyses. A p-value of less than 0.05 was considered statistically significant.

## Results

A pilot study was carried out on 18 randomly selected parents (nine fathers and nine mothers) who visited the pediatric clinic of King Khalid Hospital in AlMajma'ah, Saudi Arabia. The Cronbach’s alpha coefficient of the questionnaire was 0.79, indicating good reliability. The value of Cronbach’s alpha was more than 0.70, which is satisfactory and acceptable according to the literature [17-18].

The descriptive statistics of the respondents are shown in Table 1. Of the 1,311 eligible respondents, majority (75.1%) were mothers of the children while only 24.9% of the respondents were fathers of the children, and 29.7%, 34.4%, 24.3%, and 11.6% of the respondents had one child, two children, three children, and four or more children that were less than 14 years, respectively. Majority (76.7%) of the respondents were from the central region. Majority (87.5%) affirmed that their children did not have any chronic diseases such as asthma, diabetes, or anemia, and majority (79.6%) confirmed that the vaccination of all their children was up-to-date for age.

**Table 1:** Characteristics of respondents and their children

Variable	Frequency	Percentage (%)
Are you		
Father	327	24.9
Mother	984	75.1
Number of children less than 14 years		
One child	390	29.7
Two children	451	34.4
Three children	318	24.3
Four or more children	152	11.6
Place of residence		
Central region	1006	76.7
Eastern region	96	7.3
Western region	156	11.9
Northern region	27	2.1
Southern region	26	2
Are there chronic diseases such as asthma, diabetes, or anemia among your children?		
No	1147	87.5
Yes	164	12.5
Have all your children completed the necessary vaccinations for their age?		
No	267	20.4
Yes	1044	79.6

**Figure 1:**
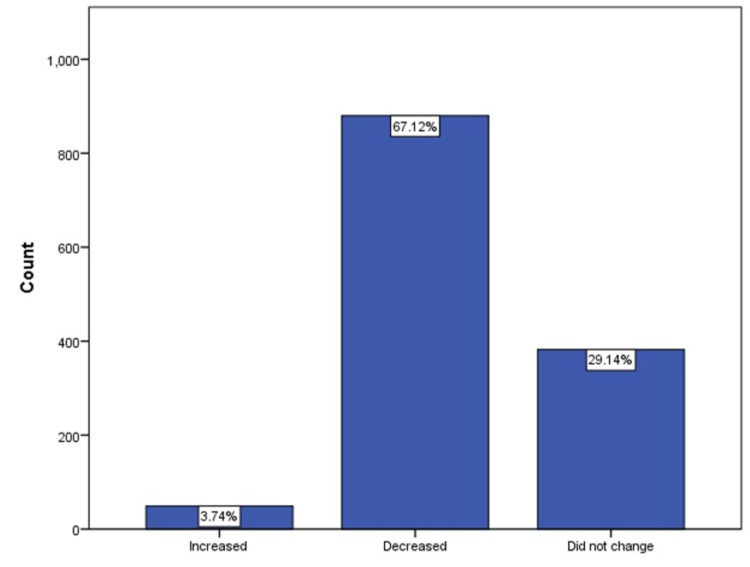
Participants’ perception of the pandemic effect on hospital visits (whether to the ER or the Clinics)

The descriptive analysis for the assessment of hospital visits during the pandemic showed that 67.1% (majority), 29.1%, and 3.7% of the respondents responded that the pandemic has reduced, did not affect, and has increased their hospital visit, respectively (Figure 1). Table 2 shows that 47.4% of the appointments at the children’s clinic were canceled or postponed either by the respondents or the hospital during the pandemic. However, majority (78.6%) of the respondents did not think that not going to the hospital either for an emergency or clinic negatively affected their children’s health and care. Similarly, only 21.7% of the respondents responded that going to the hospital during the pandemic is more dangerous than not going.

**Table 2:** Assessment of the hospital visits during the pandemic (N=1311)

Variable	Frequency	Percentage (%)
Has an appointment at your child’s clinic been canceled or postponed (by you or the hospital) during the pandemic?		
No	690	52.6
Yes	621	47.4
Do you think that the lack of going to the hospital (whether for emergency or clinic) negatively affected your child’s health and care?		
No	1030	78.6
Yes	281	21.4
Do you think going to the hospital is more dangerous than not going?		
No	471	35.9
Yes	285	21.7
Maybe	555	42.3

The descriptive analysis presented in Table 3 to assess the reasons for decreased hospital visits and its effect on pediatric care during the pandemic showed that 56.4%, 51.6%, and 19.9% of the respondents responded that lack of the need to go to the hospital, fear of contracting the virus, lack of appointments from the clinics in the hospital, respectively, were the reasons why their children did not visit the hospital. Furthermore, 13.2% of the respondents thought that the hospital was crowded, and 11.1% had difficulty going to the hospital because of the curfew.

**Table 3:** Assessment of the reasons for decreased hospital visits and its effect on pediatric care during the pandemic (multiple answers)

Variable	Frequency	Percentage (%)
What is the reason for your children’s lack of visits to the hospital (whether for emergency or clinics)?		
Lack of the need to go to the hospital	740	56.4
Fear of getting infected by the virus	676	51.6
Lack of appointments for clinics in the hospital	261	19.9
Thinking the hospital is crowded	173	13.2
Difficulty going due to curfew	146	11.1
Others	4	0.3
What is the effect of not going to the hospital (either to the emergency or to the clinic) on your children?		
No effect	879	67.0
One of my children missed an appointment to get one of his vaccinations	181	13.8
I could not consult a doctor about something, which negatively affected my child	178	13.6
Delayed diagnosis of one of my children with a certain disease	100	7.63
One of my children’s conditions deteriorated	88	6.71
One of my children ran out of treatment and we couldn’t get a cure	77	5.87

Majority (67%) claimed that not going to the hospital did not affect their children. Furthermore, 13.8% of the respondents stated that one of their children missed an appointment for vaccination, 13.6% stated that they were unable to see a doctor for the consultation of their child(ren), 7.6% of the respondent stated that not going to the hospital resulted in delayed diagnosis of a certain disease, 6.7% stated that their children’s conditions deteriorated, and 5.9% stated that a child ran out of treatment and, therefore, could not receive appropriate treatment.

Inferential statistics

Cross-tabulation results showed a significant relationship between the presence of chronic diseases such as asthma, diabetes, or anemia among children and the self-impression of the pandemic effects on hospital visits χ^2^(2, 1311) = 22.16, p < 0.001. Respondents whose children have a chronic disease such as asthma, diabetes, or anemia believed that the pandemic increased their hospital visits compared to those whose children did not have chronic diseases.

There was a significant bivariate association between the reasons for decreased hospital visits and the participants’ beliefs of the pandemic effects on hospital visits χ^2^(10, 2000) = 82.00, p < 0.001. A significant association was found between the effects on pediatric care outcomes and participants’ beliefs of the pandemic effects on hospital visits χ^2^(10, 1503) = 60.04, p < 0.001. There was a significant association between decreased hospital visits and missing an appointment for vaccination, delayed diagnosis, deterioration of participants’ children condition, and running out of treatment and inability to refill.

## Discussion

COVID-19 pandemic had a major effect on hospitals visits by the general public. This study focused on hospital visits by children less than 14 years of age in the KSA. Global data suggests clearly that people were reluctant to go even to the ED of hospitals after the announcement of the pandemic [6].

Slightly more than two-thirds (67%) of the parents responded that the pandemic has seriously jeopardized their efforts in accessing appropriate medical health care for their children even in the post-pandemic era. Our findings revealed a significant association between having children with a chronic disease and the self-reported increase in hospital visits during the pandemic. Furthermore, the main statistically significant reasons for the self-reported decline in hospital visits during the pandemic were lack of the need to visit the hospital, fear of getting infected by the virus, and inability to get appointments for clinics. When participants were asked about their opinions on the impact that resulted from the pandemic on the care of their children, majority of them reported that the decreased hospital visits due to the pandemic has not affected their children’s care and that was statistically significant. However, missing appointments for vaccination, delayed diagnosis, deterioration of children’s condition, and inability to refill a treatment were statistically significant consequences of the self-reported decline in hospital visits.

Overall, the findings of this study showed that the pandemic has seriously impacted the demand for healthcare services by children and their parents. Moreover, the findings are consistent with those in the literature that reported a sharp decline in pediatric ER visits or any other associated hospitalizations [19]. Our study reported a marked reduction in the number of visits made by the parents of children at primary healthcare clinics for routine check-ups. This is probably associated with the risk of contracting the SARS-CoV-2, and presumably, to comply with the government’s instruction for children to stay at home [20].

One of the major causes for the decrease in pediatric check-ups at primary clinics is strongly correlated with the parents’ fear that they or their child might contract the SARS-CoV-2 infection [21]. This is in line with the findings of Lazzerini et al. (2020) [22] who analyzed a dataset of 12 children who were admitted after delayed access to the healthcare facility between March 23 and March 27, 2020. Due to the delayed access, two children with type 1 diabetes developed severe ketoacidosis and were admitted to the hospital. One of the children was brought to the ER 7 days after the onset of persistent high-grade fever. One of them eventually died. Two children with symptoms of acute-onset of leukemia were brought to the ED after 7 days of high-grade fever. One had severe anemia and one died a few days after admission. A child suffering from long-lasting convulsions was brought to the hospital by the parents after three episodes of convulsion that could not be managed by the parents. The child was diagnosed with bacterial pneumonia. Similarly, a 3-year-old child was admitted to the hospital after persistent high-grade fever and was diagnosed with sepsis secondary to pyelonephritis. A neonate with hypertrophic pyloric stenosis was brought to the ER after several days and finally reached the ER in hypovolemic shock. Similarly, a 2-year-old child was taken to the ER after several days because of persistent bouts of vomiting and was diagnosed with “severe hypoglycemia.” Similarly, a child unable to pass feces was kept at home for more than a week and was eventually brought to the ER and was diagnosed with an abdominal tumor. From these 12 cases, six children were admitted to the ICU because they had critical medical conditions and four died. In all cases, the parents reported that they had been avoiding the hospital because they were afraid of contracting SARS-CoV-2 [22]. After the confirmation of the first positive case of SARS-CoV-2 infection, the government of Saudi Arabia proactively implemented the lockdown, supplemented with a curfew from 06:00 am to 07:00 pm. Even during the Eid Al-Fitr holidays, the government of Saudi Arabia imposed a stringent 24-hour curfew in all the major regions of the kingdom [23].

As the hospitals were busy working to provide medical care to the patients, most of the unessential visits and pediatric primary care appointments were either canceled or postponed. In this study, 20% of the parents reported that they were unable to get appointments for their children even in the post-COVID era. If clinical appointments are missed at the primary care level of the health system, it will lead to missed doses of vaccines, delayed presentation of children with acute medical conditions such as type 1 diabetes, and the deterioration in the management of chronic conditions such as attention deficit hyperactivity disorder (ADHD) and obesity [24].

The decreased frequency of visits, as reported by parents, was mainly because of two reasons: 1) they could not have an appointment with a doctor, 2) they (parents and kids) were afraid of contracting SARS-CoV-2. Missing an appointment with primary healthcare providers can be detrimental to the children’s health. Research has shown that missing primary healthcare visits can enormously contribute to the gaps in the routine immunization of children [25], and this can potentially lead to an increase in infections that are vaccine-preventable [26].

To the best of our knowledge, this is the first study conducted to measure the frequency of hospital visits during the pandemic in the pediatric age group in the KSA. It is the first study to assess the effects of the pandemic on hospital visits and subsequently pediatric care outcomes. Limitations of this study are represented by the small sample size, reliance on the participants’ memory, and the inability to access medical records. Therefore, further studies are needed for more accurate conclusions.

## Conclusions

This study was conducted to assess the impact of the COVID-19 pandemic on hospital visits and pediatric care outcomes in the KSA. We hypothesized that the pandemic has led to a reduction in pediatric hospital visits which might influence pediatric care outcomes.

We found that there was a decrease in the frequency of hospital visits. This decrease was attributed to the lack of the need to go to the hospital or to the fear of being infected by the virus. A significant association was observed between the presence of chronic diseases and increased hospital visits. Moreover, a significant association was found between the participants’ beliefs of the pandemic effect on hospital visits and its effect on the pediatric care outcomes. Further studies are needed for more accurate conclusions.

## Appendices

Questionnaire

1- Are you:

a. A father

b. A mother

2- Number of children less than 14 years?

a. One child

b. Two children

c.  Three children

d. Four children or more

3- Place of residence?

a. Central region

b. Eastern region

c.  Western region

d. Northern region

e. Southern region

4- Are there chronic diseases such as asthma, diabetes or anemia among your children?

a. Yes

b. No 

5- Have all of your children completed the necessary vaccinations for their age?

a. Yes 

b. No

6- What do you think about the changes in the patterns of your children visits to the hospital during the pandemic?

a. Hospital visits increased during the pandemic

b. Hospital visits decreased during the pandemic

c. Hospital visits did not change during the pandemic

7- Has an appointment at your child’s clinic been canceled or postponed (by you or the hospital) during the pandemic?

a. Yes 

b. No

8- What is the reason for your children’s lack of visits to the hospital (whether for emergency or clinics)? [You can choose more than one answer or write your specific answer]

a. Lack of the need to go to the hospital

b. Fear of getting infected by the virus

c. Lack of appointments for clinics in the hospital

d. Other (specify): …………………………………………………………………………………………………………………………………………………………………………………………………………………………………………

9- Do you think that the lack of going to the hospital (whether for emergency or clinic) negatively affected your child's health and care?

a. Yes 

b. No

10- What is the effect of not going to the hospital (either to the emergency or to the clinic) on your children? [You can choose more than one answer or write your specific answer]

a. One of my children’s condition deteriorated

b. One of my children missed an appointment to get one of his vaccinations

c. Delayed diagnosis of one of my children with a certain disease

d. Other (specify): ………………………………………………………………………………………………………………………………………………………………………………………………………………………………………………

11- Do you think going to the hospital is more dangerous than not going?

a. Yes

b. No 

c. Maybe
